# A standardized marking procedure for ENT operations to prevent wrong-site surgery: development, establishment and subsequent evaluation among patients and medical personnel

**DOI:** 10.1007/s00405-022-07448-x

**Published:** 2022-06-29

**Authors:** Christian Rohrmeier, Narmeen Abudan Al-Masry, Rainer Keerl, Christopher Bohr, Steffen Mueller

**Affiliations:** 1 Faculty of Medicine, University of Regensburg, 93042 Regensburg, Germany; 2 ENT Medicinal Office, Bahnhofstr. 19, 94315 Straubing, Germany; 3 Department of Otorhinolaryngology, St. Elisabeth Hospital, St.-Elisabeth-Str. 23, 94315 Straubing, Germany; 4 Department of Otorhinolaryngology, University of Regensburg, 93042 Regensburg, Germany; 5 Department of Oral and Maxillofacial Surgery, University of Regensburg, 93042 Regensburg, Germany

**Keywords:** Marking procedures, Wrong-site surgery, Wrong-side surgery, Patient safety, Checklist

## Abstract

**Purpose:**

Wrong-site surgeries are rare but potentially serious clinical errors. Marking the surgical site is crucial to preventing errors, but is hindered in the ENT field by the presence of many internal organs. In addition, there is no standardized marking procedure.

**Methods:**

Here, an ENT surgical-marking procedure was developed and introduced at a clinic. The procedure was evaluated through anonymized questionnaires. This study was conducted over a 6-month period by interviewing patients and, at the beginning and end of this period, doctors and other surgical staff.

**Results:**

The internal organ-marking problem was solved by applying a fixed abbreviation for each procedure onto the shoulder in addition to marking the skin surface as close to the organ as possible. The procedure was described as practicable by 100% of the interviewees; 75% of the ENT physicians and 96.3% of the other surgical staff considered the procedure highly important for preventing site confusion, and 75% of the physicians had a consequently greater feeling of safety. Of the 248 patients surveyed, 96.0% considered the marking procedure useful, and 75.8% had a consequently greater feeling of safety. For 52.0%, the marking reduced their fear of the operation.

**Conclusions:**

For the first time, a standardized procedure was developed to mark the site of ENT surgery directly, uniformly and safely on patients. The procedure was judged to be useful and practicable and was also deemed crucial for preventing site confusion. Patients felt safer and less fearful of the operation due to the marking.

## Introduction

Wrong-site surgeries (WSSs) are among the rarest but potentially worst and most shocking errors in healthcare for the public. The consequences for both the patients and the practitioners involved can be momentous, medically, legally, socially and emotionally [1–3]. WSS is an umbrella term for surgery performed on the wrong side or on the wrong body part, with the wrong procedure or on the wrong patient [1, 4].

Unfortunately, there are few solid and no robust current data on the incidence of WSS. This is mainly because, apart from information from insurance companies or through court cases, such data are mainly based on the willingness of healthcare workers to voluntarily report them [2]. A systematic review by DeVine et al. in 2010 concluded that the incidence of WSS is between 0.09 and 4.5 per 10,000 [3].

A large survey of American ENT physicians found that 6.1% of all adverse events in ENT clinics were WSSs, of which 23.1% involved the wrong organ and 15.4% the wrong side. Fifty-four percent of these errors resulted in severe morbidity [5]. In an email survey on paranasal sinus surgery, 9.3% of 455 otolaryngologists responded that they had or heard of WSS in their career, and 61% were concerned about such an error [6]. In another study, 21% of the ENT physicians surveyed reported that they had already been involved in a WSS [7].

To prevent such errors, in 1997, the American Academy of Orthopedic Surgeons was one of the first professional societies to recommend that surgeons preoperatively place their initials on the surgical site [3]. In USA, the “Universal Protocol for Preventing Wrong Site, Wrong Procedure, and Wrong Person Surgery” was introduced in 2003 by the Joint Commission (JC), the largest certification organization of healthcare facilities. This checklist, which requires a preoperative verification process, marking of the surgical site, and a team time-out immediately prior to surgery, was mandatory for all accredited hospitals as of 2004 [1, 3, 4]. Then, in 2008, the WHO Surgical Safety Checklist was published as part of the WHO’s Safe Surgery Lives campaign, which also required marking of the site to be operated on [8]. This checklist was examined in a worldwide multicenter study at eight hospitals. The introduction of the checklist resulted in a significant decrease in the postoperative death rate from 1.5% to 0.8% (*p* = 0.003), a significant decrease in inpatient complications from 11.0% to 7.0% (*p* < 0.001) and reductions in the overall rate of postoperative infections (*p* < 0.001) and unplanned reoperations (*p* = 0.047) [9]. Even after the introduction of checklists, studies showed that further improvement in the use of checklists could reduce mortality by 20% [10].

Other studies have also proven that at least half of WSSs can be prevented using such protocols [8, 11, 12]. However, it has not yet been proven with sufficient evidence that checklists significantly reduce the number of WSS cases [1, 3]. Moreover, such studies are very difficult to perform, because WSS events are rare overall and thus require a very large number of operations to be studied [1]. In addition to the preoperative checks to be carried out, improved communication through team time-outs and, above all, the marking of the intervention site are of crucial importance [8]. If possible, this should be carried out by the surgeon using a uniform marking method and confirmed by the patient [2].

In otolaryngology, there is no standardized marking technique to date [6]. This is mainly because in otorhinolaryngology, direct marking of the area to be operated on is usually not possible, as many organs, such as the tonsils, are not visible from the outside. Therefore, checklists are typically used in ENT without performing any marking. Unfortunately, since the introduction of checklists in ENT clinics, there are no robust data on whether WSSs are still a relevant problem in this specialty. Thus, no reliable statement can be made as to how much the additional introduction of a marking system would reduce WSSs.

The aim of the present study was nevertheless to develop a practicable and unambiguous marking procedure for ENT surgery and to evaluate its effect on the perception of safety among patients, surgeons, anesthesiologists and nursing staff.

## Materials and methods

### Marking procedure

A system for ENT medicine for marking surgical intervention sites was developed and subsequently introduced at a medium-sized German clinic with a large patient catchment area. At the ENT clinic, a modified WHO checklist had already been used on a mandatory basis for years; markings were voluntary and without specifications until the start of the study.

During development, the specifications were as follows:

The marking procedure should

- clearly mark the correct side, the correct organ and, if possible, the procedure to be performed;
- also work for internal organs;
- clearly mark all procedures and locations (if several procedures are to be performed on the same patient);
- be simple, highly visible and durable.

### Evaluation

The marking procedure was introduced at the clinic, tested and further improved (pen color, pen thickness, body site, etc.). After approximately 3 months, questionnaires for the surgeons, anesthetists, nursing staff and operated patients were prepared and distributed. The questionnaires were reviewed in advance by medical personnel, particularly with regard to the appropriateness and relevance of the questions. This expert panel considered the validity of the questionnaires in terms of their content and design to be appropriate.

The questionnaires for the patients were distributed over a period of 6 months, and the medical staff received identical questionnaires at the beginning and end of this period. The printed questionnaires consisted of closed questions (single- and multiple-choice questions, as well as matrix questions), and there was also an open question for suggestions for improvement.

### Statistics

Statistical analysis and graphical representation were carried out using Microsoft Excel 2016 software for Windows (Microsoft Corporation), SPSS Statistics 24 software (IBM Corporation, Armonk/USA) and EvaSys V8.0 software (evasys GmbH, Lüneburg, Germany). The median test was used to compare the results of the groups surveyed.

## Results

### Marking procedure

**Fig. 1:**
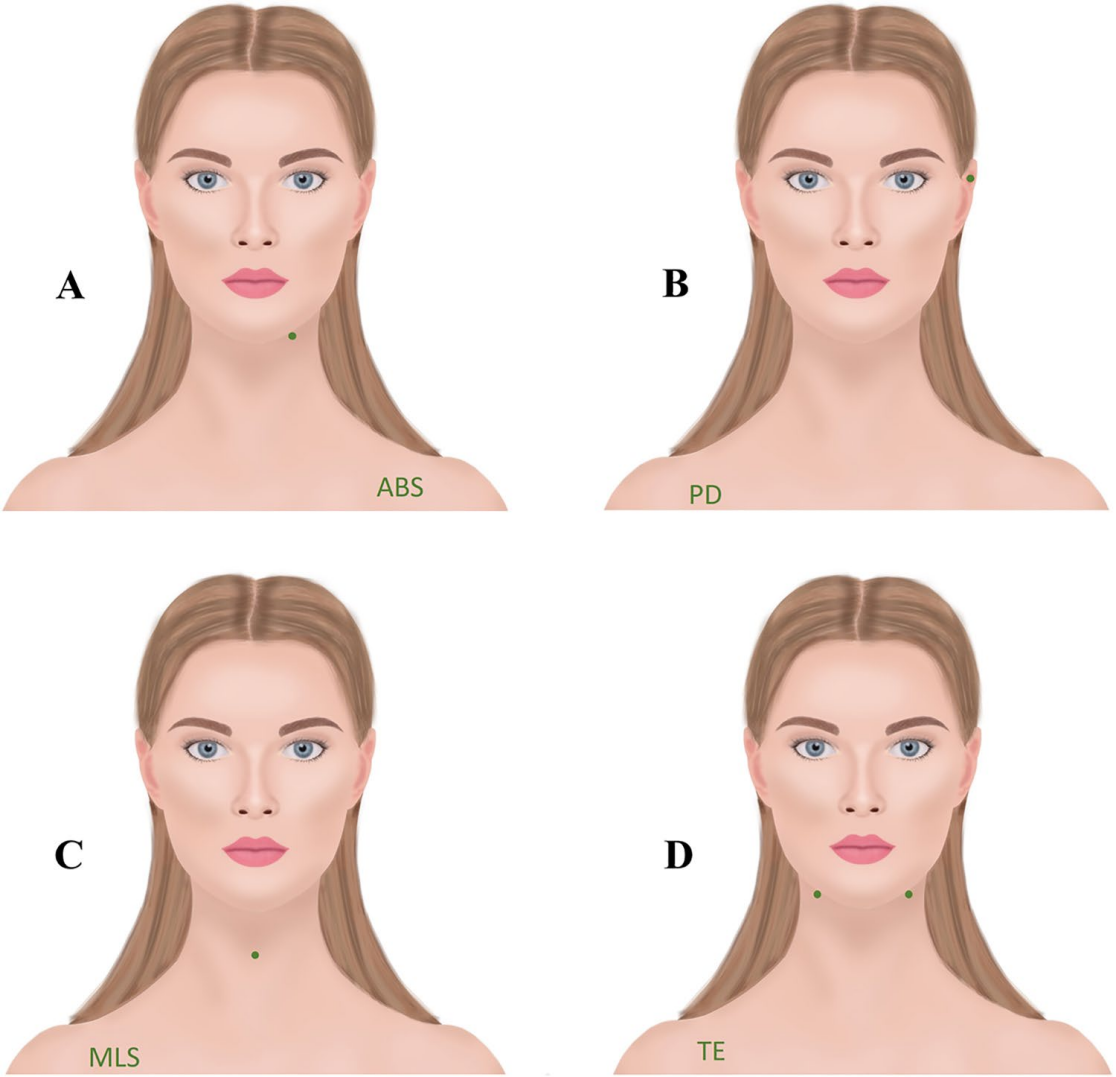
Examples of the marking procedures. For each procedure, the abbreviation defined for it is applied to a shoulder; in addition, the location of the procedure is marked by a thick dot. For internal organs, the marking is performed as close as possible to the skin surface. *A* Abscess of the floor of the mouth (ABS) on the left, *B* Tympanic drainage (PD) on the left, *C* Microlaryngoscopy (MLS), and *D* Tonsillectomy (TE) on both sides

In otorhinolaryngology, external marking of surgical sites alone is usually not sufficient, as many organs, such as the vocal folds, cannot be marked from the outside. This problem was solved by applying a standardized text defined for each procedure (see Table 1) to one of the two patient shoulders. In practice, it does not matter which side of the shoulder is used. This text describes the operation (OP) to be performed, and the site of the operation is then additionally marked with a thick dot as close as possible to the relevant organ (for examples, see Fig. 1).

**Table 1:** List of abbreviations

Intervention	Abbreviation
Abscess	ABS
Adenotomy	AT
Anterolateral femoral flap	OS.LAP
Cochlear implant	OHR
Conchotomy	CONCHO
Epistaxis	EPI
Palatoplasty/uvuloplasty	ORO
Ear canal cholesteatoma	OHR
Skin tumor removal	HAUT 1/X, 2/X…
Hypopharyngeal diverticulum	DIVERT
Laryngectomy	LE
Lateral neck cyst	ZYSTE
Latissimus dorsi flap	LAT.LAP
Eyelid surgery	LID
Lymph-node extirpation	LK
Median neck cyst	ZYSTE
Microlaryngoscopy	MLS
Nasal bone repositioning	NBR
Paranasal sinus surgery	NNH
Nasopharyngeal tumor resection	TU
Neck dissection	ND
Orbital fracture	ORBITA
Oro-/hypo-/pharyngeal tumor resections for carcinoma	TU
Otopexy	OHR
Panendoscopy	PAN
Paracentesis	PC
Parotidectomy	PAROTIS
Tympanic drainage	PD
Pectoralis flap	PEC.LAP
Platysma flap	PLAT.LAP
Provox system	PROVOX
Radial lobe	RAD.LAP
Rhinoplasty	RP
Septoplasty	SPL
Salivary gland endoscopy	ENDO
Stapes surgery	OHR
Submandibulectomy	SUBMAN
Thyroidectomy/hemithyroidectomy	STRUMA
Tonsillectomy	TE
Tonsillotomy	TO
Tracheostomy/tracheostoma closure	TRACH
Lacrimal surgery	TW
Tympanoscopy/tympanoplasty Type I or III	OHR

The table shows the standardized abbreviations defined for each intervention. These are then plotted on one of the two shoulders. (The list was translated into English, and the abbreviations are partly based on German operation designations.)

**Fig. 2:**
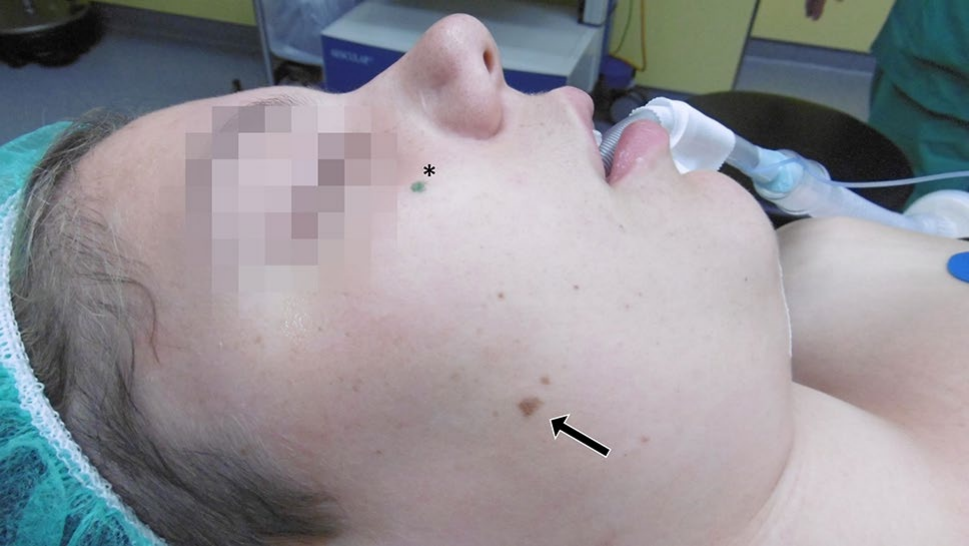
Risk of confusion in skin lesions. A green marker has proven to be most useful for marking, as green provides a clear contrast to natural skin changes. In this example, the arrow (→) indicates a skin change, and the star (*) indicates the green pen mark

A green, non–water-soluble marker with a line thickness between 1.5 and 3 mm (e.g., Edding 3000 Permanent Marker) has proven to be most useful for marking. Green was chosen, because it provides a clear contrast to natural skin changes. Particularly with black pens, there could be some ambiguity in this respect (see Fig. 2).

**Fig. 3:**
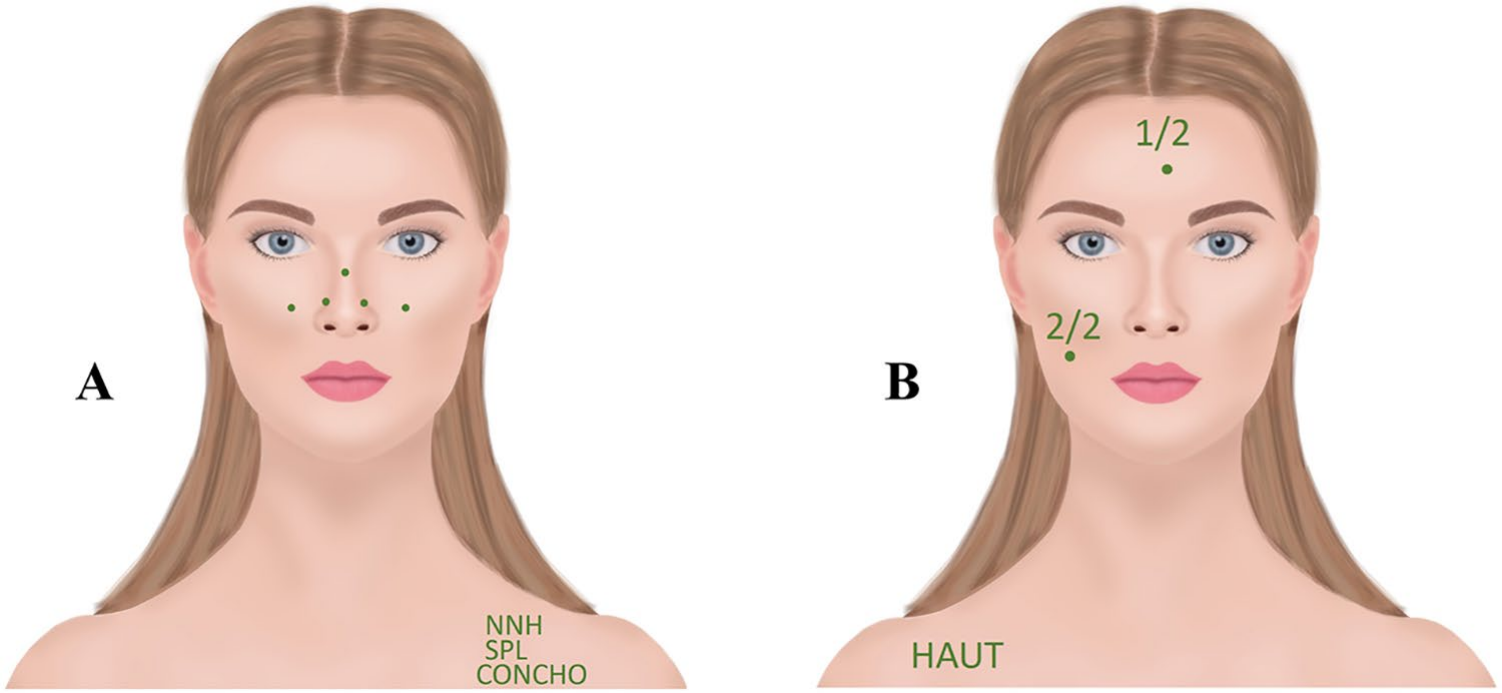
Multiple procedure sites. *A* In the case of multiple intervention sites, all sites are marked by a dot, and each intervention is additionally marked by an abbreviation on the shoulder: in the example, a paranasal sinus operation (NNH) with septoplasty (SPL) and conchotomy (CONCHO). *B* For skin tumors (HAUT), the number of tumors is also indicated

In the case of multiple intervention sites, all sites are marked by a dot, and each intervention is marked by an abbreviation on the shoulder. For skin tumors, the number of tumors is also indicated (see Fig. 3).

Marking must be done by a doctor, if possible, at patient admission or on the ward, if this is not possible in the operating theater sluice. It is important that the marking is performed before administering premedication, as the patient must be awake and fully conscious to confirm the procedure and the surgical site.

Before entering the operating theater, a final check is made to see whether the marking is present. This is documented and checked by a WHO checklist modified and expanded for use in the clinic. Transport into the operating theater is not permitted without marking.

### Results of the questionnaires

From February to July 2021, 248 operated patients were interviewed. Questionnaires of 13 parents of operated children and 3 legal guardians were excluded. During the study period, another 27 patients were not marked (especially emergency operations), not interviewed, and consequently not included. A total of 17.7% of those operated on were over 67 years, and 56.9% were male.

At the beginning and end of the same period, 12 ENT surgeons and 27 other hospital staff involved in the operation (6 anesthetists, 10 technical assistants, 6 nurses and 5 anesthesia nurses) were interviewed anonymously using standardized questionnaires.

### Patients

**Fig. 4:**
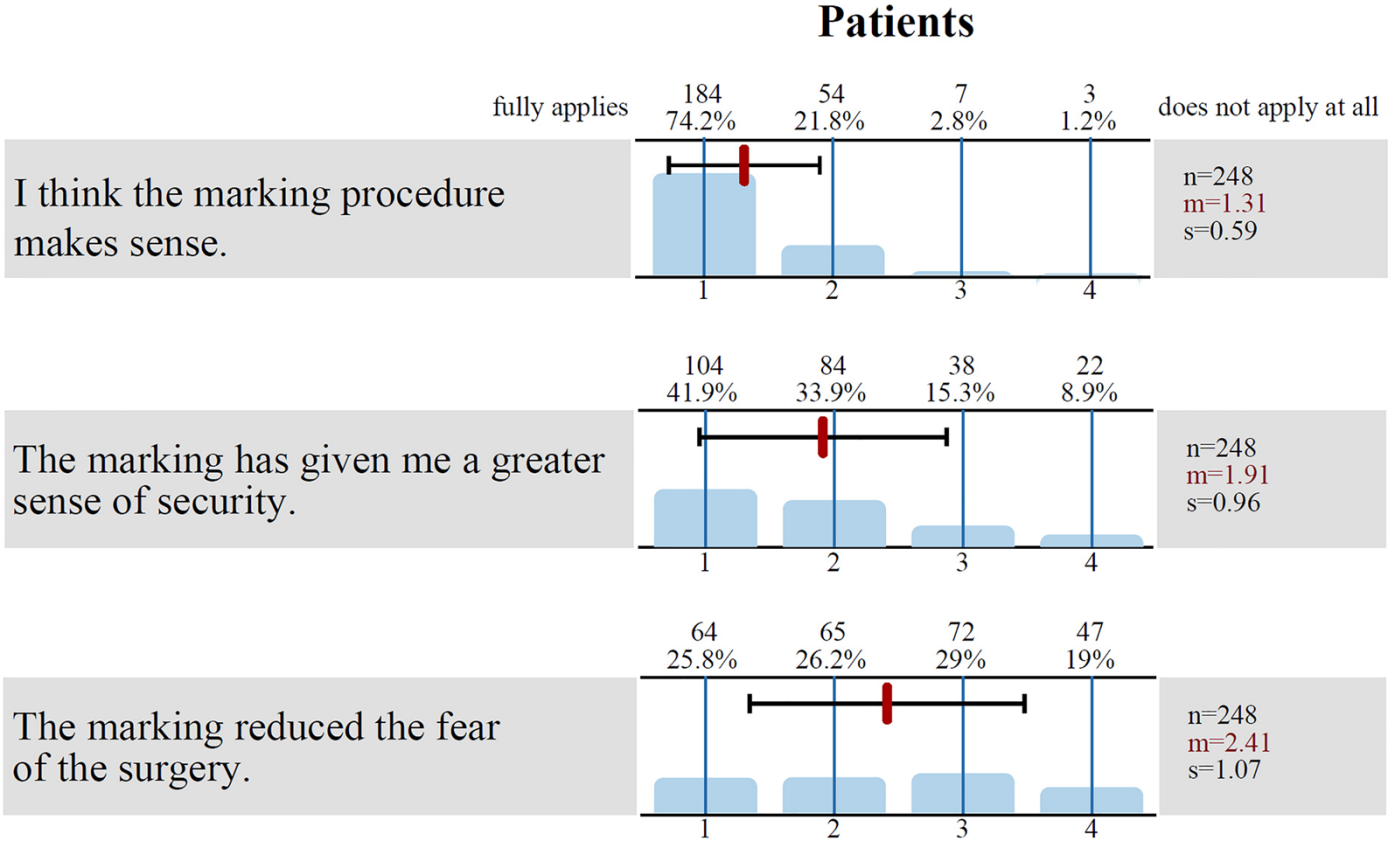
Results of the patient survey. The graph shows the results of the patient survey for three different questions. The bars show the distribution of the answers (*1* fully applies, *2* mostly true, *3* often does not apply, and *4* does not apply at all), and above the bars are the absolute (*n*) and percentage frequency of the answers. The black line shows the standard deviation (*s*), and the red line shows the mean value (*m*)

A total of 96.0% of patients agreed with the statement “I think the marking procedure is useful”, and 75.8% agreed with the statement that “the marking […] gave me a greater sense of security”. The fear of the operation had been reduced by the marking for 52.0% (see Fig. 4), and only 2.4% had been frightened by the marking itself.

For 76.6% of patients, the purpose of the marking procedure had been explained in detail. A total of 81.5% had confirmed the procedure and side of the operation to the doctor before the marking, and 10.9% stated that this had not been the case.

Only 7.8% stated that the marking on the face had been disturbing.

Free comments included the following: “Please explain before and not after”, “A little more explanation, especially when explaining the abbreviation. […]”.

### ENT surgeons

**Fig. 5:**
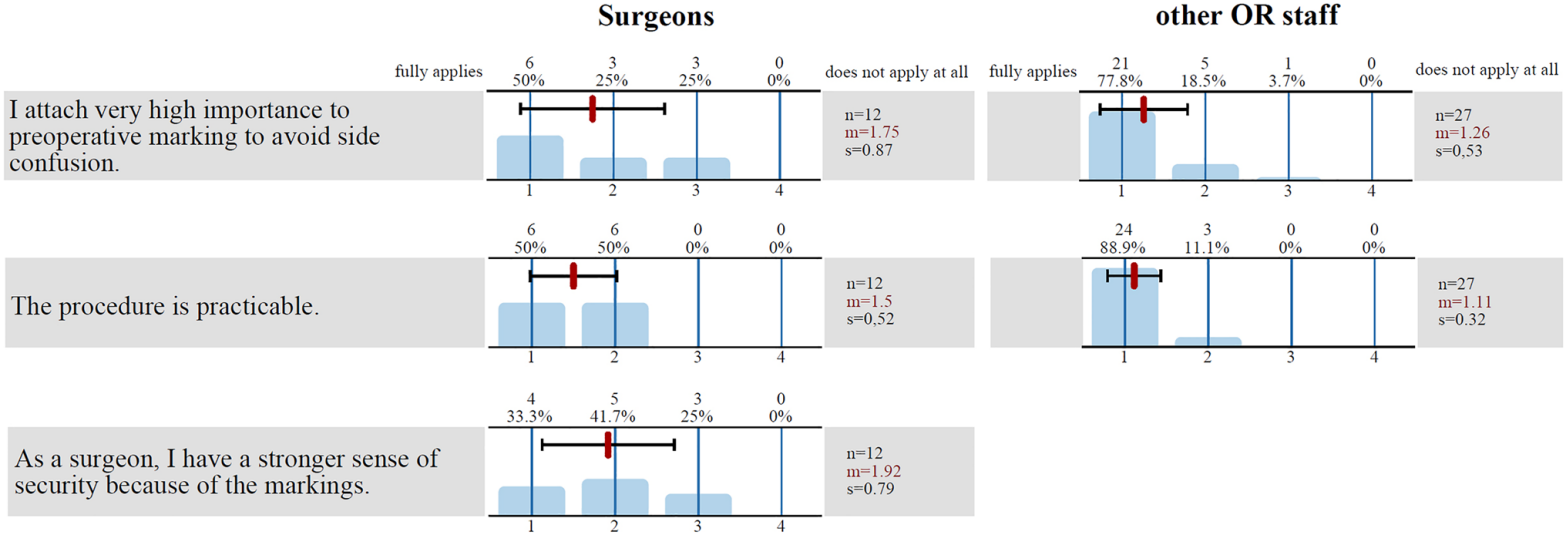
Results of the surgeons and OR staff survey. The graph shows the results of the survey of the surgeons and the other OR staff regarding three different questions (at the end of the 6 months). The bars show the distribution of the answers (*1* fully applies, *2* mostly true, *3* often does not apply, and *4* does not apply at all), and above the bars are the absolute (*n*) and percentage frequency of the answers. The black line shows the standard deviation (*s*), and the red line shows the mean value (*m*)

At the beginning of the 6-month period, 91.7% (75.0% when surveyed at the end of the 6-month period) of ENT surgeons stated that they placed a high value on preoperative marking to prevent side confusion. A total of 66.7% (75.0%) would have a stronger feeling of safety as a result (see Fig. 5).

91.7% (100%) of the ENT surgeons stated that they were well instructed in the procedure, all (100%) found it practicable, and 91.7% (91.7%) found it logically structured. A total of 91.7% (91.7%) stated that they had explained the meaning of the marking to the patients in each case, which took less than 1 min for 83.3% (91.7%).

A total of 83.4% (66.7%) checked for the presence of the marking preoperatively, and 58.3% (58.3%) checked before the skin incision. A total of 91.7% (91.7%) confirmed the correctness of the marking with the patient; 100% (91.7%), with the admission protocol; 83.3% (75.0%), with the operating room schedule; and 8.3% (16.7%), with colleagues. A total of 58.3% (66.7%) of the doctors reported having performed markings in the ward to date; 66.7% (66.7%), at registration for outpatient surgery; 75.0% (83.3%), at the theater sluice; 58.3% (75.0%), in the anesthesia holding room; and 58.3% (50.0%), in the theater. Sixty-seven percent (66.7%) would need less than 1 min for marking, and the remaining 33.3% (33.3%) would need between 1 and 5 min. Only children under 10 years of age refused to be marked.

For emergencies, 8.3% (25.0%) of the surgeons would never mark, 50.0% (41.7%) would rarely mark, 25.0% (16.7%) would sometimes mark and 16.3% (16.3%) would often mark.

Free comments included the following: “Mark children and disabled persons only in the presence of the supervisors, not only in the anesthesia holding room.”

### Other OR staff

In total, 92.6% (96.3%) of the OR staff surveyed said that they placed a high value on preoperative marking to prevent side confusion.

A total of 44.4% (81.5%) said that they had been well instructed in the procedure, and all (100%) found it practicable.

A total of 85.2% (96.3%) checked for the presence of the marking preoperatively.

In total, 85.2% (77.8%) checked for the correctness of the marking with the patient, 51.9% (66.7%) checked it with the admission protocol, 70.4% (70.4%) checked it with the operating room schedule and 18.5% (29.6%) confirmed it with colleagues. Free comments included the following: “All patients should be marked on the ward” and “Increase the convenience of surgeons.”

### Differences between ENT surgeons and other OR staff

The answers to identical items in the questionnaires were compared between the group of ENT surgeons and the other OR staff. There were no significant differences. The difference was greatest for the question of whether the presence of the marking was checked preoperatively, but this was also not significant, with *p* = 0.179 (*p* = 0.054 in the last survey).

## Discussion

A study of surgical malpractice claims found that such instances of malpractice mostly occur in routine operations with experienced surgeons. Seven percent of the errors investigated in the study were WSSs [13]. An analysis of court cases between 1995 and 2010 in England found 137 cases in the field of otology and neurotology, of which 6 were related to WSS. Interestingly, most of the trials involved tympanostomy tubes or myringotomies, 14 in total and 4 of the 6 WSS cases [14]. This shows that confusion is not unique to complex procedures. It should be noted that court cases only show the tip of the iceberg, and approximately 97% of negligently injured patients do not seek compensation [14]. Additionally, the degree of possible physical disability due to treatment errors plays only a minor role. There is no correlation to the patient's perception of the harm and thus to their anger and sense of broken trust [15].

Most of the time, it is not just a single error that leads to WSS. It is usually a confluence of several active errors and weaknesses in the system [15]. A good theoretical model for this is that of the Swiss cheese by James Reason [8]: this shows layers of cheese (= defensive mechanisms) with holes, where each layer provides protection against potential errors. The more layers and the smaller and rarer the gaps are, the more likely it is that errors that occur will be detected and prevented. Checklists represent such layers. The two most commonly used surgical checklists worldwide, sometimes in modified form, are the JC’s Universal Protocol (UP) and the WHO’s Surgical Safety Checklist (SSC) [16, 17].

According to the SSC, the marking of the surgical site should be performed by the surgeon himself or herself. Marking of midline organs (e.g., thyroid) or single organs (e.g., spleen) is not necessarily needed, but overall uniform marking of all cases is recommended as a safety check [18]. Additionally, according to the UP, markings should be performed by the surgeon who is ultimately responsible for the procedure and is present at the surgery. However, delegation is allowed under certain circumstances. Moreover, the marking must be clear and consistent. Exceptions to direct marking include mucosal surfaces and procedures with minimal access to internal organs, percutaneously or via a natural orifice [16]. Both checklists recommend involving the patient in the verification process and marking process.

Now, it is the case that the exceptions mentioned in the protocols in the ENT area represent the rule, since most organs, such as vocal folds, tonsils or adenoids, are not visible from the outside. Data from Shah et al. confirmed the difficulty in ENT using the example of paranasal sinus surgery [6]. A survey of American ENT physicians showed that less than 50% of these physicians mark, although the risk for WSS, particularly due to laterally inverted CT images, is potentially increased in paranasal sinus surgery. The study also showed that in surgeries where confusion had occurred, more than 60% had not been marked.

Although it is difficult to objectively investigate safety strategies due to the relative rarity of WSS events [1], studies and interviews have shown that marking the surgical site is an important building block and thus a crucial layer in the cheese model for preventing WSS [1, 3, 8, 11, 12, 19]. There is thus a need for a safe, simple and unambiguous ENT labeling procedure [2, 15]. To the best of our knowledge, the present study was the first to develop and implement such a standardized procedure for direct marking of procedure sites in ENT surgery at a hospital.

The problem of marking internal organs was solved by not only marking on the surface of the skin as close to the organ as possible with a thick green dot but also by additionally applying an abbreviation firmly defined for the operation on one of the two shoulders. The use of an “X” was deliberately avoided, as such a mark could also mean that surgery should not be performed at this location. In other studies, a “yes”, scratch marks by a needle, arrows or the surgeon’s initials are described as marking signs [2, 11]. Since a marking procedure should not only be unambiguous but also uniform, the dot seemed an appropriate choice. To prevent possible confusion with skin patches, green was specified as the marking color. The abbreviation on the shoulders not only defined the organ more precisely but also indicated the procedure. For example, “TE” was applied for a tonsillectomy and “TO” for a tonsillotomy.

After the introduction of the marking procedure developed in this study at an ENT clinic, an evaluation was carried out by means of questionnaires. All ENT surgeons and all operating theater staff surveyed found the procedure practicable. The time required for marking was also manageable.

Of the ENT surgeons, three quarters had a stronger feeling of safety due to the markings. Interestingly, initially, 91.7% of ENT surgeons still considered the marking procedure to be of great importance in preventing side confusion, whereas after 6 months, only 75% of them did. The remaining OR staff saw it differently: here, the percentage rose by 3.7% to 96.3% after the study. Although not significant, the difference between surgeons and the remaining staff was even more pronounced when it came to checking for the preoperative presence of the markings (66.7% of the surgeons vs. 96% of the remaining staff).

Such differences between the different professional groups in the OR have also been shown by studies on OR checklists. For example, a study from Finland described that anesthetists and nurses used the introduced WHO checklist more often than ENT physicians; furthermore, there were complaints that ENT physicians would partly neglect it [20]. Additionally, in the present survey, one piece of feedback was that the convenience of the surgeons needed to be increased. Another study on safety in the operating theater showed that reports of near-miss incidents come least often from the surgeons [19].

Potential explanations for why surgeons are less open to measures such as checklists or tagging are probably including an already high workload due to bureaucracy, time pressure and lack of staff [21]. Overconfidence and lack of awareness of a possible source of error could also play a role. A Cochrane analysis showed that targeted educational intervention (in a study of dentists) had a significant effect on reducing WSSs [1]. The awareness of errors seemed to increase as a result.

A total of 91.7% of the ENT surgeons confirmed the correctness of the marking with the patient; over 90%, with the admission protocol; approximately 80%, with the OR plan; and approximately 10%, with colleagues. The remaining OR staff showed a similar result. It should be noted that the clinic staff was aware that a study was being conducted, which could represent bias. Whether such high rates are maintained in normal clinical routines cannot be predicted but should be a goal.

One's own memory and the asking of colleagues are not reliable controls. This was shown in a study by Pikkel et al., who asked ten ophthalmic surgeons about the side to be operated on before each operation. Only 73% were able to name the correct side on the basis of the patient's name alone, and only 83% were able to name the correct side when they made eye contact with the patient. The age and experience of the surgeon did not play a role. There was a significant correlation between the number of wrong side decisions and the number of operations on the same day and the time interval to the preliminary examination [22].

Matching with the OR plan alone also poses a risk. Studies have identified misreporting in the OR schedule as a source of error for WSSs [19, 23]. The surest confirmation lies in reconciling multiple sources, especially the inclusion of the not-yet-sedated patient in combination with the admission/informed consent document [11, 12, 16, 18]. Relying solely on the information provided by the patient or family members can also be a cause of WSS [1, 3].

If the clinical structure does not allow marking with the patient’s assistance before the operation, then an alternative by means of a form would be conceivable. Knight et al. studied this approach in over 112,500 patients. The operating site was already marked on a form together with the patient during the information session and signed by the patient; later, the marking on the patient was carried out by the operating theater nurses on the basis of this document. There was only one confusion of sides [24]. However, marking by the patient himself or herself did not prove to be reliable; one-third of the patients did not participate [15].

The marking procedure developed in this study was considered useful by 96.0% of the patients, and three quarters had a greater feeling of safety as a result. Slightly more than half of the patients stated that the marking reduced their fear of surgery, while only a few were scared (2.4%) or disturbed (7.8%) by the marking. This can be described as a win–win situation both for the safety of the clinical procedure and for the psychological stress of the patients. However, in the case of questionnaires, the potential influence of the wording of the question on the respondent's answer must be considered.

The developed marking method can represent organs and types of surgery, but there are certain limitations. In some cases, the exact nature of the operation only becomes clear in the course of the operation; for example, a hearing-improving operation may develop into stapesplasty or tympanoplasty. The result of a frozen section histology can also have a considerable influence on the development of the planned operation. However, most crucial to the safety achieved, whether by marking procedures or checklists, is the care with which they are performed and thus the human factor. No procedure alone should be regarded as a magic bullet for preventing WSS. Only a combination with redundant communication, integration of the entire surgical team into the safety precautions and, of course, direct contact between surgeon and patient can provide the greatest possible safety by reducing the size and number of holes in the Swiss cheese model [24]. Increasing the reliability of safety measures (team time-out, checklists, etc.) is crucial to everyday clinical practice, as doing so reduces the size of the holes in the cheese and the number of layers required. Each layer involves additional effort and time.

All safety levels should be adapted as best as possible to the respective clinic and should also be as short, simple and situation-specific as possible [25]. It should be communicated to all involved that not only checklists and specifications but also communication and process safety are in the foreground of surgical practice. The goal should be an atmosphere of effective communication and a culture of safety [10].

## Summary

In the present study, a standardized procedure was developed for the first time to mark intervention sites for ENT operations directly, uniformly and safely on patients. The procedure was introduced at a clinic and evaluated as useful and practicable by patients, surgeons and other operating theater staff. The procedure was considered to be of great importance in preventing side confusion. Moreover, the patients had a greater feeling of safety and less fear of the operation due to the marking. However, future studies must show whether the additional marking has a relevant effect on the number of WSSs compared with checklists without marking.
